# Estimation of Symptom Severity Scores for Patients with Schizophrenia Using ERP Source Activations during a Facial Affect Discrimination Task

**DOI:** 10.3389/fnins.2017.00436

**Published:** 2017-08-03

**Authors:** Do-Won Kim, Seung-Hwan Lee, Miseon Shim, Chang-Hwan Im

**Affiliations:** ^1^Department of Biomedical Engineering, Chonnam National University Yeosu, South Korea; ^2^Psychiatry Department, Ilsan Paik Hospital, Inje University Goyang, South Korea; ^3^Department of Biomedical Engineering, Hanyang University Seoul, South Korea

**Keywords:** schizophrenia, electroencephalogram (EEG), generalized linear model (GLM), electrophysiological biomarker, psychiatric diseases

## Abstract

Precise diagnosis of psychiatric diseases and a comprehensive assessment of a patient's symptom severity are important in order to establish a successful treatment strategy for each patient. Although great efforts have been devoted to searching for diagnostic biomarkers of schizophrenia over the past several decades, no study has yet investigated how accurately these biomarkers are able to estimate an individual patient's symptom severity. In this study, we applied electrophysiological biomarkers obtained from electroencephalography (EEG) analyses to an estimation of symptom severity scores of patients with schizophrenia. EEG signals were recorded from 23 patients while they performed a facial affect discrimination task. Based on the source current density analysis results, we extracted voxels that showed a strong correlation between source activity and symptom scores. We then built a prediction model to estimate the symptom severity scores of each patient using the source activations of the selected voxels. The symptom scores of the Positive and Negative Syndrome Scale (PANSS) were estimated using the linear prediction model. The results of leave-one-out cross validation (LOOCV) showed that the mean errors of the estimated symptom scores were 3.34 ± 2.40 and 3.90 ± 3.01 for the Positive and Negative PANSS scores, respectively. The current pilot study is the first attempt to estimate symptom severity scores in schizophrenia using quantitative EEG features. It is expected that the present method can be extended to other cognitive paradigms or other psychological illnesses.

## Introduction

Since patients with schizophrenia have their own unique signs and symptoms that generally show dramatic changes over the progress of the illness or during treatment, it is of great importance to precisely evaluate the symptom severity of each patient. Although the assessment of symptom severity in schizophrenia is critical in establishing successful treatment strategies or evaluating the effectiveness of treatments, only few quantitative diagnosis tools exist to evaluate the symptom severity of individual patients with schizophrenia (Lakhan, 2006).

Thus far, symptom severity in schizophrenia has been generally diagnosed based on interview-based tests performed by trained psychiatrists. Although the inter-rater reliability or test-retest reliability of these criteria is reported to be high (Kay et al., 1987; Bell et al., 1994), it is still possible that the results might be highly influenced by initial assessment (Mortimer, 2007) or subjective opinions of the psychiatrists (Norman et al., 1996). Moreover, diagnosis of the negative symptoms of schizophrenia is relatively more difficult (Lindström et al., 1994; Norman et al., 1996) than that of positive symptoms, which are based on evident symptoms. Patients with negative symptoms might not show any evident signs or may often show general behaviors overlapping with other mental illnesses (Andreasen, 1982). Therefore, the development of quantitative methods to diagnose schizophrenia or evaluate symptom severity in individual patients can potentially help to enhance the diagnostic accuracy of schizophrenia.

Over the past several decades, researchers have put great effort into investigating predictable characteristics of schizophrenia. For instance, several studies have reported the possibility of diagnosing schizophrenia based on either functional (Sabeti et al., 2007, 2009) or structural characteristics (Takayanagi et al., 2011) of the brain. Various studies have also shown that electrophysiological measures such as P300 (Shenton et al., 1989; Strik et al., 1993; Kim et al., 2014), auditory steady-state response (ASSR) (Spencer et al., 2008; Shin et al., 2011), loudness-dependent auditory evoked potential (LDAEP) (Ostermann et al., 2012; Wyss et al., 2013), and mismatch negativity (MMN) (Hirayasu et al., 1998; Javitt et al., 2000; Youn et al., 2003) are strongly correlated with symptom severity in schizophrenia and thus might be used as trait or state markers of the disease. There is a growing consensus that the precise estimation of symptom severity is of great necessity as it can be used to predict quality of life (Bow-Thomas et al., 1999), long-term outcome (Harrison et al., 1996; Ho et al., 1998), relapse (Birchwood et al., 1989), and functional outcomes (Mueser et al., 1991; Bowie et al., 2008); however, to the best of our knowledge, no study has yet investigated how accurately such electrophysiological biomarkers can predict symptom severity of each individual patient.

In this preliminary study, we investigated whether it is possible to accurately predict the symptom severity of patients with schizophrenia from electrophysiological measurements. We used event-related potential (ERP) and its source imaging results as candidate variables for the estimation of symptom severity of patients with schizophrenia. Electroencephalography (EEG) was recorded while patients performed a facial affect discrimination task that was introduced in our previous study (Kim et al., 2013). We found that patients with schizophrenia not only showed significantly decreased neuronal activations during facial affective processing compared to normal controls, but also showed significant correlations between such neuronal activations and Positive and Negative Syndrome Scale (PANSS) scores (Kim et al., 2013). Based on these findings, we established a mathematical prediction model based on electrophysiological biomarkers to estimate the Positive/Negative PANSS scores of individual patients.

## Materials and methods

We recruited a total of 23 patients with schizophrenia for the current study. All participants were stable, right-handed, with normal or corrected-to-normal vision. The mean age of the participants was 32.2 ± 10.1 (mean ± SD) years, and 11 of them were female (Table 1). All participants had been diagnosed with schizophrenia based on the Structured Clinical Interview for Diagnostic and Statistical Manual of Mental Disorders, 4th Edition (DSM-IV), Axis I Psychiatric Disorders (Kay et al., 1987). All subjects were taking atypical antipsychotics (olanzapine, *n* = 11; risperidone, *n* = 12). This study was carried out in accordance with the recommendations of the Institutional Review Board (IRB) of Inje University Ilsan Paik Hospital with written informed consent from all subjects. All subjects gave written informed consent in accordance with the Declaration of Helsinki. The protocol was approved by the IRB of Inje University Ilsan Paik Hospital.

**Table 1:** Demographic data and symptom ratings from 23 patients with schizophrenia.

	**Schizophrenia (*n* = 23)**
Age (years)	32.2 ± 10.1
Male, female	12, 11
Education duration (years)	12.8 ± 2.1
Number of hospitalizations	1.7 ± 1.4
Duration of illness (years)	5.2 ± 4.9
Antipsychotic drug dosage (mg)	391.30 ± 97.30
PANSS total score	81.8 ± 25.8
Positive scale	20.2 ± 7.8
Negative scale	18.7 ± 7.4

**Figure 1:**
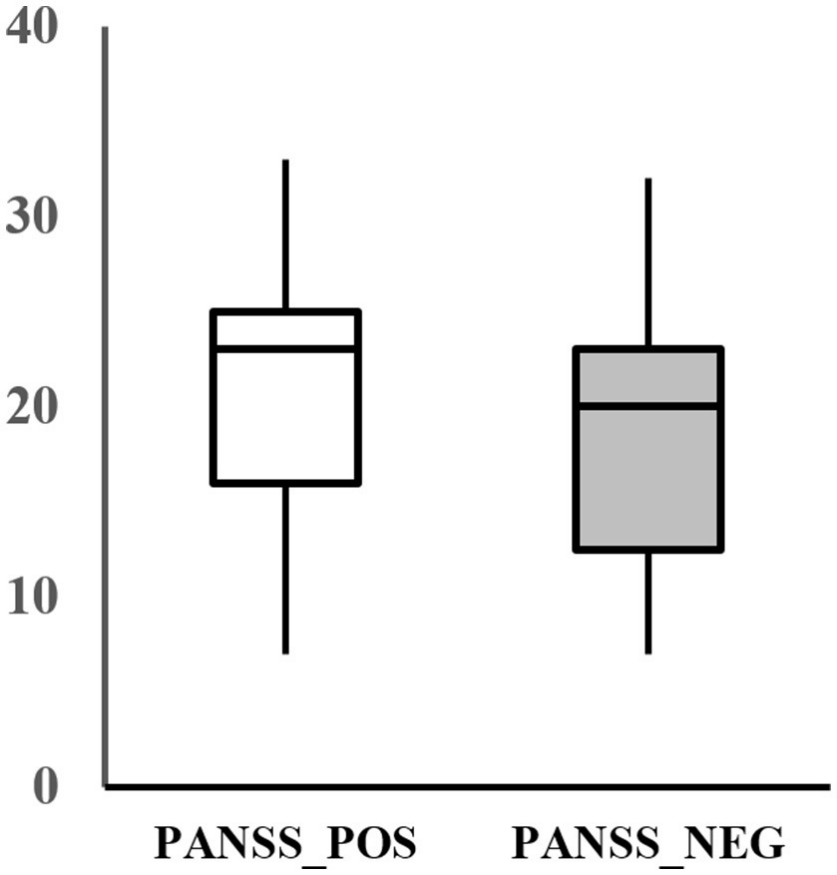
Positive (PANSS_POS) and negative PANSS score (PANSS_NEG) distribution of the subjects.

The assessment of the psychiatric symptom of the patients was done by a trained psychiatrist using the PANSS. The PANSS evaluates the severity of the two common types of symptoms (positive and negative) in schizophrenia as well as the general psychopathology of the patient based on the interview as well as reports of family members. The positive scale is rated by seven positive symptoms such as hallucination and delusion, which refer to an excess or distortion of normal functions. The negative scale is also rated by seven items of negative symptoms, which show loss or reduced functions compared to healthy subjects (e.g., emotional withdrawal). Each item is rated from 1 (absent) to 7 (extreme) and summed up for each category, resulting in a scale of 7–49 for each symptom scale. Additionally, the general scale of the patient is rated by 16 items that evaluates the general psychopathological symptoms, such as anxiety, tension, and poor attention. The average positive and negative score of the patients recruited for this study was 20.2 ± 7.8 and 18.7 ± 7.4, respectively (Figure 1).

Neural source activations were evaluated using EEG data recorded from 64 electrodes including two electrooculography channels (VEOG and HEOG). A total of 288 facial stimuli were presented to each patient in a randomized order, with an equal presentation probability with respect to three different emotional conditions (neutral, fear, and happy) (Lee et al., 2004). In this study, we only used EEG data from the neutral face condition in order to reduce the dimensionality of features because a greater number of candidate features does not always guarantee more precise prediction results.

**Figure 2:**
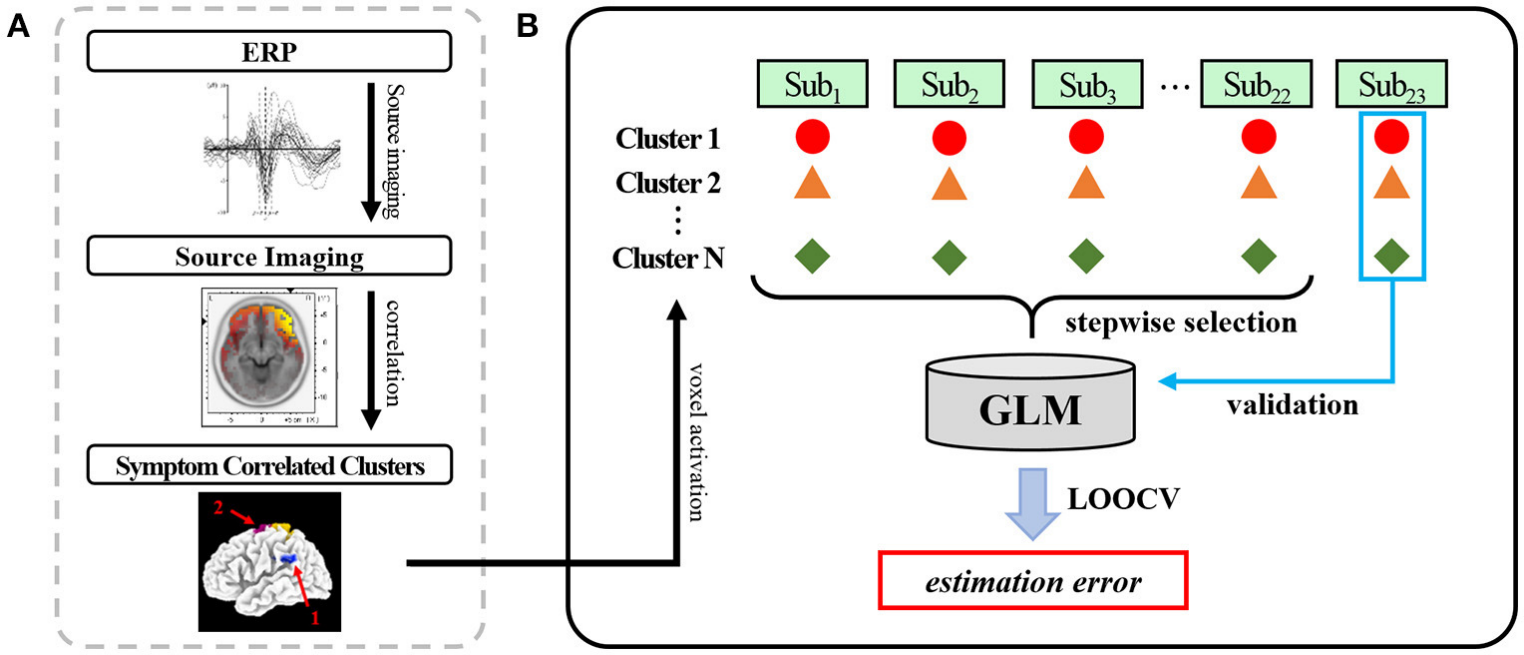
An illustration of the overall analysis procedure: **(A)** Using the identified face-related ERP components (P100, N170, N250, P300), the source activation of each ERP was estimated using sLORETA. Multiple nearby voxels showing significant correlation between source activation and symptom severity scores were clustered. **(B)** The voxel activation with the highest correlation within each cluster was used as an independent variable of the general linear model to estimate the symptom severity score. The generated model was validated using leave-one-out cross validation (LOOCV).

As the first step of the analysis, we obtained source activation of distinct brain areas which has a significant correlation with the symptom score (Figure 2A) The overall analysis procedure is illustrated in Figure 2. The recorded EEG signals were preprocessed to eliminate unwanted artifacts and averaged to identify four ERP components known to be associated with facial emotion processing: P100, N170, N250, and P300 (Lee et al., 2010; Kim et al., 2013). The source image for each ERP component was reconstructed using sLORETA (Pascual-Marqui, 2002) for each patient. The sLORETA is one of the most widely-used source imaging methods that showed reliable source estimates even in the presence of noise or in low-density EEG configurations (Babiloni et al., 2010; Bae et al., 2011; Saavedra et al., 2012). In our previous study that used the same patient data, we have shown that source activation of N170 estimated using sLORETA was decreased in patients with schizophrenia compared to normal controls in multiple brain areas (Jung et al., 2012). After the execution of sLORETA software, voxels with a significant correlation with symptom scores were identified (Table 2). The significant voxels were then clustered based on anatomical location, and the voxel with the maximum correlation within each cluster was selected. The detailed procedures for data acquisition and data preprocessing are well described in our previous study (Kim et al., 2013).

**Table 2:** Brain regions showing significant correlation between PANSS scores and ERP source activation during the neutral face stimulus condition.

**PANSS type**	**Emotion type**	**ERP**	**Cluster No**.	***r***	**Structure (Brodmann area)**	**MNI coord. of maximum voxel**		
						***X***	***Y***	***Z***
Positive	Neutral	P100	1	−0.647	Inferior parietal lobule (BA 40)	−50	−35	35
			2	−0.639	Precentral gyrus (BA 6)	−15	−20	70
			3	−0.662	Precuneus (BA 31)	−15	−50	35
			4	−0.616	Insula (BA 13)	40	−45	20
		N170	5	−0.607	Middle frontal gyrus (BA 10)	35	60	−5
		N250	6	−0.657	Medial frontal gyrus (BA 10)	20	45	0
Negative	Neutral	P100	1	−0.702	Sub-gyral (BA 37)	−45	−45	−15
			2	−0.693	Middle temporal gyrus (BA 39)	−50	−75	15
		N250	3	−0.600	Middle frontal gyrus (BA 10)	30	50	0

*Maximum correlation values (r) and their MNI coordinates are listed for each cluster*.

To estimate each patient's positive and negative symptom severity, we established a prediction model based on the general linear model (GLM) (Henderson et al., 2001; Coan and Allen, 2004). Each patient's ERP amplitudes and the source activation of the voxel that had the maximum correlation value in each cluster were selected as independent variables in the regression model (Figure 2B). The source activation was the standardized score obtained from sLORETA. The Positive and Negative scales of the PANSS were used as dependent variables in the regression model. To exclude unnecessary or relatively less relevant variables, a stepwise selection method was applied with entrance/exit tolerances of 0.05/0.10 (*p*-values).

## Results

The prediction model of the Positive scale of the PANSS was composed of two predictors [source activations at the medial frontal gyrus (N250) and the precentral gyrus (P100)]. The weighted linear combination of these predictors was significant [*F*_(2, 22)_ = 35.402, *p* < 0.001] and explained approximately 78% of the variance in the Positive scale (*R* = 0.883, *R*^2^ = 0.780). A prediction model of the Negative scale of the PANSS was constructed using the source activations of the sub-gyral (P100) and middle frontal gyrus (N250), which was also significant [*F*_(2, 22)_ = 17.507, *p* < 0.001] and explained approximately 63% of the variance in the Negative scale (*R* = 0.798, *R*^2^ = 0.636). The selected predictor variables of each model are listed in Table 3. Note that we also considered ERP amplitudes as candidate predictor variables, but none of them was included in the final prediction models as the ERP amplitudes were not selected by the stepwise selection method with the entrance tolerance of *p* = 0.05 at all. Even when we changed the entrance tolerance to *p* = 0.5 to construct a regression model only with ERP amplitudes, the best prediction models to estimate positive and negative PANSS scores could explain only 20.8 and 13.9% of the variances in each score, respectively. Likewise, we also considered age of patients as an independent variable of the model, but it was not included in the final prediction model.

**Table 3:** Constructed prediction models and their validation results.

**PANSS**	**Variables**	***b***	**SE-b**	**β**	**Pearson's r**	**sr^2^**	**Structure coefficient**	***p***	**Leave-one-out cross-validation (mean ± std)**
Positive	Constant	32.064	1.636						3.34 ± 2.40
	Medial frontal gyrus	−4.840	0.834	−0.611	−0.657	0.371	−0.744	<0.001^***^	
	Precentral gyrus	−1.719	3.060	−0.592	−0.639	0.348	−0.724	<0.001^***^	
Negative	Constant	29.179	2.015						3.90 ± 3.01
	Sub-gyral	−7.646	1.960	−0.561	−0.702	0.277	−0.880	0.001^**^	
	Middle frontal gyrus	−1.910	0.679	−0.405	−0.600	0.144	−0.752	0.014^*^	

* p < 0.05;

** p < 0.01;

*** *p < 0.001*.

To evaluate the performance of the prediction models, we adopted a leave-one-out cross-validation (LOOCV) strategy. LOOCV is the most extreme and accurate type of cross-validation strategy to estimate prediction errors where every data sample is used in turn for the validation of the model constructed with the remaining data (Hastie et al., 2003; Ko et al., 2011). LOOCV results showed that the symptom scores could be predicted with an average error of 3.34 ± 2.40 and 3.90 ± 3.01 for the Positive and Negative scales, respectively (Table 3).

For comparison, each patient's Positive and Negative scale scores were assumed to have either the average or median of the group symptom scores. Mean errors were 6.43 ± 4.18 (Positive) and 6.03 ± 4.05 (Negative) when the average symptom scores were used, and 6.09 ± 5.48 (Positive) and 5.96 ± 4.36 (Negative) when the median symptom scores were used, both of which were much higher than the scores estimated with our prediction models.

## Discussion

In this study, we investigated whether the symptom severity scores of individual patients with schizophrenia could be estimated using ERP current source activations during facial affect processing. We found that Positive and Negative scale scores can be estimated with a fairly high accuracy considering the wide range of each score (0~49), demonstrating the possibility of a quantitative assessment of psychiatric symptoms based on electrophysiological biomarkers. To the best of our knowledge, the current preliminary study is the first attempt to quantitatively estimate clinical symptom severity of schizophrenia from electrophysiological biomarkers.

The evaluation of a patient's psychiatric symptom scores using quantitative biomarkers provides a raw estimate of the patient's symptom severity without the need for an interview with psychiatrists or clinical psychologists. Specifically, this technique would be useful in estimating the symptom scores of patients who have low cognitive profiles or demonstrate unreliable behavior. Further, this technique can be used as an auxiliary tool for long-term longitudinal tracking of symptom changes in order to evaluate the responsiveness of psychopharmacological treatment and to establish an appropriate patient-specific treatment strategy.

In our study results, both Positive and Negative scales could be successfully estimated because abnormal facial affect processing is regarded as one of the key symptoms of schizophrenia associated with both positive and negative symptoms (Mandal et al., 1999; Kohler et al., 2003; Hofer et al., 2009). This suggests that the selection of proper symptom-related tasks is important in building reliable prediction models. For instance, we expected that an altered P300 amplitude and its source activation would be good candidate predictors of symptom scores because they have also been frequently reported to be strongly correlated with symptomatic scores of patients with schizophrenia (Shenton et al., 1989; Strik et al., 1993; Kim et al., 2014). In addition, other (electro-) physiological biomarkers (e.g., graph theory-based indices or behavioral outcomes) may potentially improve the overall estimation accuracy. Therefore, further studies are needed to compare the performance of various tasks and/or biomarkers in terms of estimation accuracy. We expect that our approach to building a prediction model to estimate symptom severity in schizophrenia from electrophysiological biomarkers can also be applied to other psychiatric diseases such as Alzheimer's disease, depression, and bipolar disorder, using specific paradigms reflecting each disease's unique signs or symptoms.

## Author contributions

DK was responsible for the analyzing procedure wrote the manuscript. SL designed the study and wrote the protocol. MS analyzed the EEG data and produced ERP waveforms. CI supervised the study process and manuscript writing. All authors contributed to and have approved the final manuscript.

### Conflict of interest statement

The authors declare that the research was conducted in the absence of any commercial or financial relationships that could be construed as a potential conflict of interest.
